# Comparative safety of artemether-lumefantrine and other artemisinin-based combinations in children: a systematic review

**DOI:** 10.1186/1475-2875-12-385

**Published:** 2013-11-01

**Authors:** Oluwaseun Egunsola, Kazeem A Oshikoya

**Affiliations:** 1 Academic Division of Child Health, University of Nottingham, Derbyshire Children’s Hospital, Derby DE22 3DT, UK; 2 Department of Pharmacology, Lagos State University College of Medicine, IkejaLagos state, Nigeria

**Keywords:** Artemether-lumefantrine, Adverse event, Paediatrics, Children, Safety

## Abstract

**Background:**

The purpose of the study was to compare the safety of artemether-lumefantrine (AL) with other artemisinin-based combinations in children.

**Methods:**

A search of EMBASE (from 1974 to April 2013), MEDLINE (from 1946 to April 2013) and the Cochrane library of registered controlled trials for randomized controlled trials (RCTs) which compared AL with other artemisinin-based combinations was done. Only studies involving children ≤ 17 years old in which safety of AL was an outcome measure were included.

**Results:**

Four thousand, seven hundred and twenty six adverse events (AEs) were recorded in 6,000 patients receiving AL. Common AEs (≥1/100 and <1/10) included: coryza, vomiting, anaemia, diarrhoea, vomiting and abdominal pain; while cough was the only very commonly reported AE (≥1/10). AL-treated children have a higher risk of body weakness (64.9%) than those on artesunate-mefloquine (58.2%) (p = 0.004, RR: 1.12 95% CI: 1.04-1.21). The risk of vomiting was significantly lower in patients on AL (8.8%) than artesunate-amodiaquine (10.6%) (p = 0.002, RR: 0.76, 95% CI: 0.63-0.90). Similarly, children on AL had a lower risk of vomiting (1.2%) than chlorproguanil-dapsone-artesunate (ACD) treated children (5.2%) (p = 0.002, RR: 0.63, 95% CI: 0.47-0.85). The risk of serious adverse events was significantly lower for AL (1.3%) than ACD (5.2%) (p = 0.002, RR: 0.45, 95% CI: 0.27-0.74).

**Conclusion:**

Artemether-lumefantrine combination is as safe as ASAQ and DP for use in children. Common adverse events are cough and gastrointestinal symptoms. More studies comparing AL with artesunate-mefloquine and artesunate-azithromycin are needed to determine the comparative safety of these drugs.

## Background

Malaria, caused by *Plasmodium falciparum*, is an important public health problem in Africa. It accounts for an estimated 660,000 deaths in 2010, mostly among African children [1]. Due to the increasing resistance of malaria parasite to older anti-malarial drugs, such as chloroquine and sulphadoxine-pyrimethamine, artemisinin-based combination therapy (ACT) has been recommended by the World Health Organization (WHO) [2]. This approach involves the combination of artemisinin, or one of its derivatives, with other anti-malarials, such as amodiaquine, lumefantrine or sulphadoxine-pyrimethamine. Several studies have demonstrated the efficacy of ACT for malaria treatment [3]. A combination therapy explores the synergistic or additive effect of two or more drugs with different mechanisms of action to improve the therapeutic efficacy, increase the therapeutic drug life and delay the development of resistance to each of the component drugs in the combination [4].

Artemether-lumefantrine (AL) is one of the most commonly used combinations in sub-Saharan Africa. It is the first-line treatment for uncomplicated malaria in several countries. It is available in oral form as a fixed dose combination (20 mg artemether and 120 mg lumefantrine) and given as a six dose regimen for the treatment of uncomplicated malaria. Artemether has a short half-life of 2-3 hours, while lumefantrine has a half-life of about 5 days [4-6]. Artemether rapidly and extensively reduces parasite biomass, while lumefantrine clears the residual parasites from the body [7].

Although AL is widely prescribed, several other combinations are increasingly being explored and used but data are lacking on their safety. Most of the previous studies have compared the efficacies of AL and other artemisinin-based combinations, but little or no attention has been given to their safety. AL has been shown to be relatively safe when compared with other antimalarials such as quinine, sulphadoxine-pyrimethamine and chloroquine [8]. Adverse effects, such as headache, dizziness, abdominal pain anorexia, anaemia, arthralgia, myalgia, diarrhoea, vomiting, nausea, weakness and rash, have been documented [9,10]. Given the wide range of ACT available for malaria treatment and their potential adverse effects, it is imperative to compare their safety profiles. This systematic was, therefore, performed to compare the safety profiles of AL with other ACT in children.

## Methods

### Search strategy

Embase (from January 1974 to April 2013), Medline (from January 1946 to April 2013) and the Cochrane library of registered controlled trials for randomized controlled trials (RCTs) were searched for RCTs comparing AL with other artemisinin-based combinations. Search terms such as artemisinin or artemether or artesunate or dihydroartemisinin were combined with lumefantrine or amodiaquine or sulphadoxine-pyrimethamine or mefloquine or chloproguanil or dapsone or piperaquine or azithromycin. Only studies involving children ≤ 17 years old in which safety of AL was an outcome measure were included. There was no restriction on the language of publication.

### Data extraction

Two reviewers extracted data from the included studies onto data extraction form.

Data extracted from each study included: the comparator drug, the number of participants in each arm of the study, year of the study, duration of follow-up, age of the participants, dose of drugs administered, the number of deaths recorded, the number of participants who withdrew from the study, and the adverse event (AE) data for both AL and the comparator drugs. All data were compared and agreed to by both reviewers.

### Data quality assessment

To minimize the risk of bias, the quality of included randomized controlled trials was assessed using the Cochrane collaboration’s tool for assessing risk of bias in randomized trials [11]; quality of reported safety studies was also assessed using the modified CONSORT checklist for reporting of harmful effects [12]. Articles with modified CONSORT score of ≥6 out of 9 criteria were considered to have provided good quality safety reporting. All RCTs were included for meta-analyses. Highly biased and poor quality studies were subsequently excluded in sensitivity analysis. Two reviewers independently scored and agreed on articles included. Kappa statistic was used to determine the level of agreements on the quality assessment between the two reviewers (k = 0.92) [13].

### Data collection and statistical analysis

The relevant data were extracted onto an excel spread sheet. All studies were grouped based on the comparator drug and AEs in each pool were aggregated for meta-analysis. The adverse event profile of AL was used as the reference against which other artemisinin-based combinations were compared. Meta-analysis was done using Revman version 5. Relative risk was calculated for all AEs reported in more than one study. A relative risk (RR) >1 indicates a higher risk of AE in AL relative to the comparator. The pooled RR was calculated using the fixed effect model for homogenous data (I^2^ ≤50% or Chi^2^ p ≥ 0.05) and random effect model for heterogeneous data (I^2^ > 50% or Chi^2^ p < 0.05) as suggested by DerSimonian and Laird [14].

## Results

**Figure 1:**
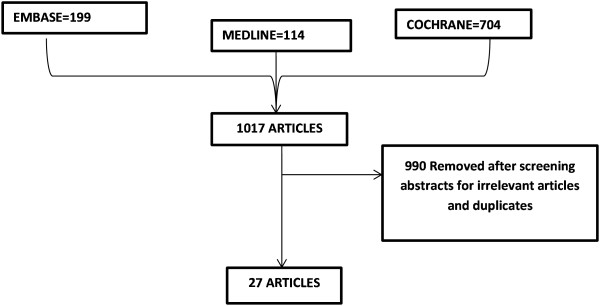
Flow chart for studies included in the systematic review.

Safety information was extracted from 27 RCTs (Figure 1), which met our inclusion criteria [15-41]. After risk of bias assessment, 7 studies were of high risk [23-25,34-37]. The age of the patients ranged between six weeks and 18 years (Table 1). In all studies, a six-dose regimen of AL (20 mg artemether and 120 mg lumefantrine) was administered over three days. The total number of children administered ACT was 15,119. Four thousand, seven hundred and twenty six AEs were recorded in 6,000 patients receiving AL. All but one of the RCTs, were conducted in Africa (Nigeria, Ghana, Benin, Uganda, Kenya, Tanzania, Ivory Coast, Burkina Faso, Senegal and Gabon). The only non-African country was Paupa New Guinea. Common AEs (≥1/100 and <1/10) among patients receiving AL included: coryza, vomiting, anaemia, diarrhoea, vomiting and abdominal pain; while cough was the only very commonly reported AE (≥1/10) (Table 2). The respiratory and gastrointestinal tracts were the most commonly affected systems constituting 35% and 33% respectively, of all the reported AEs in AL treated children. Other artemisinin based combinations compared with AL were: artesunate-amodiaquine (ASAQ), dihydroartemisinin-piperaquine (DP), chlorproguanil-dapsone-artesunate (ACD), artesunate-mefloquine (AM) and artesunate-azithromycin (AAZ).

**Table 1:** Characteristics of treatment groups

**Drugs**	**AL and DP**	**AL and ACD**	**AL and ASAQ**	**AL and AM**	**AL and AAZ**
Age range(months)	6-168	6-180	1.5-120	6-216	6-59
Median (IQR) duration of follow-up (days)	35(22.8-51.8)	35(12.3-42)	28(21-42)	28	42
Number of studies	11	3	13	2	1
Number of patients in AL arm	3343	1861	3054	239	132
Number in comparator arm	4284	1505	2964	237	129

**Table 2:** Organ-system classification and risk of adverse events in AL treated children

**System organ class**	**Adverse event**	**Number of adverse events**	**Risk (per 1000 patients)**
Gastrointestinal disorders	Abdominal pain	187	31.2
	Anorexia	442	73.7
	Diarrhoea	371	61.8
	Nausea/vomiting	471	78.5
	Others	109	18.2
General disorders	Weakness	372	62
	Pyrexia	372	62
	Mouth ulcer	13	2.2
	Salivation	7	1.2
	Unable to suck	5	0.8
	Body pain	10	1.7
	Hypersensitivity reaction	3	0.5
	Puffy face	1	0.2
Respiratory disorders	Cough	1152	192
	Coryza	449	74.8
	Dyspnoea	4	0.7
	Others	55	9.2
Central Nervous system disorders	Headache	228	38
	Dizziness	18	3
	Insomnia	23	3.8
	Convulsion	3	0.5
	Nystagmus	1	0.2
Skin and appendages disorders	Rash	55	9.2
	Pruritus	90	15
	Urticaria	2	0.3
	Others	35	5.8
Haematological disorders	Anaemia	157	26.2
	Haemolysis	3	0.5
	Thrombocytopaenia	9	1.5
	Neutropenia	42	7
Liver and biliary system disorders	Jaundice	3	0.5
	Elevated ALT	23	3.8
	Hepatomegaly	4	0.7
Musculo-skeletal system disorders	Joint pain	1	0.2
Cardiovascular disorders	Palpitation	6	1

### AL and DP

Eleven studies involving 5958 children compared AL with DP [15-25]. There were three cases of death in each of the drug groups; none was related to the drugs. The risk of serious adverse events (SAEs) was lower with AL (0.5%) than DP (1.2%), although this difference was not statistically significant (p = 0.09, RR 0.62 95%CI 0.36-1.07). In one of the studies, two unnamed SAEs in AL arm and four in DP group were considered to have causal relationship with the drugs [20]. In another study, one patient receiving AL developed severe anaemia, which was considered to be possibly associated with the drug [22]. In Table 3, the children receiving AL had a minimally higher risk of abdominal pain (10.9%) than those receiving DP (10.4%) (p = 0.09, RR 1.31, 95% CI 0.99-1.73). After a study with a high risk of bias [24] was excluded in a sensitivity analysis, the risk of abdominal pain was statistically significant (p = 0.04). Table 3 shows that the risks of other common adverse events such as pruritus, diarrhoea, cough, vomiting and weakness of the body were not significantly different between the two drug groups (p > 0.05).

**Table 3:** Relative risk of AEs between AL and other artemisinin-based combinations

**Relative risk of AEs between AL and DP**					
**Adverse event**	**Risk AL (%)**	**Risk comparator (%)**	**Relative risk (95% CI)**	P value (*significant)	**References**
Vomiting	10.3	9.7	0.99[0.86-1.13]	0.85	15-24
ǂAnaemia	3.7	10.1	0.45[0.20-1.02]	0.05	18, 20
Abdominal pain	10.9	10.4	1.31[0.99-1.73]	0.06	17, 19, 21, 24
Diarrhoea	13.3	12.6	1.03[0.89-1.20]	0.65	15-18, 20-24
Pruritus	5.8	4.5	1.28[0.81-2.02]	0.30	17, 19, 21, 24
Weakness	16.5	13.3	1.14[0.94-1.39]	0.19	15, 17, 19, 21, 24
Cough	39.8	39.2	0.99[0.92-1.05]	0.65	15, 17-19, 20-22, 24
Anorexia	12.3	11.0	1.07[0.93-1.24]	0.38	15, 17, 19-22
SAE	0.5	1.2	0.62[0.36-1.07]	0.09	15-22
**Relative risk of AEs between AL and ASAQ**					
Abdominal pain	14.5	14.3	0.81[0.46-1.40]	0.45	26, 28, 29, 31-34
Pruritus	8.4	9.5	0.78[0.54-1.11]	0.17	26, 28, 31, 34
Anorexia	14.3	17.6	0.88[0.76-1.03]	0.11	20, 26, 29, 31
ǂAnaemia	8.9	19.1	0.61[0.19-1.90	0.39	20, 30, 31, 33
Headache	13.5	7.9	1.29[0.89-1.87]	0.17	29, 32, 34
Vomiting	8.8	10.6	0.80[0.66-0.97]	*0.02	20, 26, 28-31, 33, 34
Weakness	12.8	13.4	0.84[0.63-1.13]	0.25	28, 29, 31, 34
Diarrhoea	12.0	10.4	1.09[0.89-1.32]	0.41	20, 27, 32, 34
Cough	31.4	31.2	1.01[0.91-1.12]	0.84	20, 27, 30, 32, 33
SAE	0.8	1.5	0.54[0.27-1.05]	0.07	20, 30-32
**Relative risk of AEs between AL and ACD**					
ǂAnaemia	2.8	7.2	0.35[0.09-1.43]	0.15	26, 38
Anorexia	11.8	17.8	0.80[0.63-1.00]	0.05	20, 26
Cough	29.2	23.0	1.13[0.96-1.34]	0.15	20, 26
Diarrhoea	6.0	8.6	1.28[0.96-1.73]	0.11	20, 26
Vomiting	1.2	5.2	0.63[0.47-0.85]	*0.002	20, 26, 38
SAE	1.3	5.2	0.45[0.27-0.74]	*0.002	26, 38
**Relative risk of AEs between AL and AM**					
Weakness	64.9	58.2	1.12[1.04-1.21]	*0.004	39, 40
Abdominal pain	1.7	3.8	0.44[0.14-1.41]	0.17	39, 40
Vomiting	21.3	22.8	0.75[0.27-2.05]	0.57	39, 40

ǂ Random effect model.

* (<0.05) statistically significant’.

### AL and ASAQ

Thirteen studies involving 6018 children comparing AL with ASAQ were included and analysed [20,26-37]. The risk of SAEs was not significantly different for both treatment groups (p = 0.07 RR: 0.54, 95% CI: 0.27-1.05). The percentage risks of SAEs in the AL and ASAQ groups were 0.8% and 1.5% respectively. Most of the reported SAEs were judged to be unlikely related to the medications studied. However in one study [20], four unnamed SAEs in patients receiving ASAQ and two in those receiving AL were considered to be drug related. There were only three documented cases of mortality, two in the AL group and one in the ASAQ group. None of the deaths was treatment related. Table 3 shows that the risk of vomiting was significantly lower in patients on AL (8.8%) than ASAQ (10.6%) (p = 0.002, RR: 0.80, 95% CI: 0.66-0.97). After excluding one poor quality RCT [34] in a sensitivity analysis, the risk of vomiting remained statistically significant (p=0.002). The risk of other AEs, such as weakness, pruritus, anaemia, diarrhoea, abdominal pain, anorexia and cough, was not statistically significant (p > 0.05) with or without biased RCTs.

### AL and ACD

Three studies comparing AL with ACD involving 3366 children were included in this review [20,26,38]. Two of these studies compared more than one artemisinin-based combination. All included RCTs were rated as low risk. The overall risk of SAE was significantly lower for AL (1.3%) than ACD (5.2%) (p = 0.002, RR: 0.45, 95% CI: 0.27-0.74). Forty-six (73%) and three (20%) of the SAEs in the ACD and AL groups, respectively, were due to oxidative haemolysis. Majority of the haemolytic AEs in the ACD group (56.5%) occurred in G6PD deficient children. All the three cases of haemolysis in the AL group were in G6PD normal children. There were three and four cases of deaths, respectively, for AL and ACD; none was considered treatment related. In Table 3, the risk of vomiting was significantly lower for AL (1.2%) than ACD (5.2%) (p = 0.002, RR: 0.63, 95% CI: 0.47-0.85). No other adverse event showed a statistically significant difference between both drugs.

### AL and AM

Two studies involving 476 children compared AL with AM [39,40]. None of the studies reported death or SAEs for both drugs. In Table 3, pooled analyses of both RCTs showed a statistically significantly higher risk of weakness for AL treated children (64.6%) than AM (58.2%) (p = 0.004, RR: 1.12 95% CI: 1.04-1.21). Vomiting and abdominal pain, which were the other two AEs reported in both studies, were not significantly different in both treatment groups.

### AL and AAZ

Only one RCT involving 261 patients compared the safety of AAZ with AL in paediatric patients [41]. This study showed that children taking AAZ have a significantly higher risk of vomiting than those on AL (p = 0.02). The risk of other AEs such as: dizziness, convulsion, respiratory and dermatological events were not significantly different in both treatment groups.

## Discussion

This review identified cough as the most common adverse event in children receiving AL (Table 2). Other common adverse events are: abdominal pain, vomiting, anaemia, headache and diarrhoea. These findings have also been reported by other authors [9,42]. Many of the AEs were adjudged to be symptoms of malaria or signs of a progression of the disease. Generally, AL was well tolerated with only a few SAEs recorded. Only two unnamed SAEs were considered to be related to AL treatment. AL treated patients showed a significantly lower risk of vomiting compared with ASAQ, ACD and AAZ. A higher risk of weakness was observed in AL treated children compared with AM.

Due to increased risk of haemolysis, the clinical development of ACD, which is a very effective anti-malarial drug, was terminated at phase III [43]. A multicentre RCT by Premji et al. [38] has demonstrated a higher incidence of haemolysis in children receiving ACD than AL. Given the high prevalence of glucose 6-phosphate dehydrogenase (G6PD) deficiency (up to 30%) in sub-Saharan Africa [44], and the fact that G6PD deficient children are prone to the haemolytic effect of the oxidant metabolite of dapsone [45], halting the development of ACD for malaria treatment in Africa was justified. Malderen et al. [46] showed that G6PD deficient children treated with ACD have a higher, but non-statistically significant risk of having haemolysis and a >2 g/dl drop in their haematocrit, than non-deficient patients. Although ACD development has been stopped, it may still be relevant in the future fight against malaria. If such time ever comes, treatment would have to be individualized, with rapid test of G6PD status done before ACD prescription. Such tests have been approved by the US Food and Drug Administration for warfarin therapy and colon cancer treatment [47,48].

Although mefloquine is associated with neuropsychiatric symptoms such as nightmares, hallucinations and anxiety [49-51], none was recorded in the two RCTs involving AM in this review. The small number of patients involved in both studies may not be adequately powered to detect this event. Gastrointestinal events were the most common adverse events in patients on AM. This is similar to the report from a study in Nigeria [52]. None of the gastrointestinal events however showed a significant difference between the patients on AM and AL. Treatment with AL, more than AM, significantly increased the risk of weakness of the body.

Some of the meta-analyses were limited by the relatively small number of patients in the comparator groups. Therefore, caution is required when extrapolating the results to the general population. Besides, such small sample size may not be sufficiently powered to detect rare events. Several of the RCTs reported adverse events rather than adverse reactions of the antimalarial drugs. This made it impossible to determine the causal relationship between the antimalarial drugs and the AEs. It was, therefore, difficult to determine whether an AE is symptomatic of the disease or drug related. In some other studies, safety reporting was either selective or inadequate, with some authors failing to indicate the severity of AEs. Some of these limitations have been identified in studies evaluating the quality of safety reporting in RCTs [12,53].

## Conclusion

Artemether-lumefantrine combination is as safe as ASAQ and DP for use in children. Common adverse events are cough and gastrointestinal symptoms. Haemolysis in G6PD deficient children makes the use of ACD undesirable. The few numbers of studies comparing AL with AM and AAZ would not enable us to make a firm conclusion on their comparative safety; therefore, more studies are needed to determine the comparative safety of AL with AM and AAZ.

## Abbreviations

AAZ: Artesunate-azithromycin; ACD: Chorproguanil-dapsone-artesunate; ACT: Artemisinin combination therapy; AE: Adverse event; AL: Artemether-lumefantrine; AM: Artesunate-mefloquine; ASAQ: Artesunate-amodiaquine; CI: Confidence interval; CONSORT: CONsolidated Standards of Reporting Trials; DP: Dihydroartemisinine-piperaquine; G6PD: Glucose-6-phosphate dehydrogenase; RCT: Randomized controlled trial; RR: Relative risk; SAE: Serious adverse event; WHO: World Health Organization.

## Competing interests

The authors declare that they have no competing interests.

## Authors’ contributions

OE and KAO conceived the idea and planned the research; OE performed the database search; OE and KAO reviewed the retrieved papers for eligibility and performed the data extraction; OE and KAO performed the risk of bias assessment; OE performed the meta-analyses; OE wrote the first draft; KO edited and reviewed the manuscript; OE prepared the final draft. Both OE and KAO agreed to the final draft of the manuscript.
